# Vgll3 and the Hippo pathway are regulated in Sertoli cells upon entry and during puberty in Atlantic salmon testis

**DOI:** 10.1038/s41598-018-20308-1

**Published:** 2018-01-30

**Authors:** Erik Kjærner-Semb, Fernando Ayllon, Lene Kleppe, Elin Sørhus, Kai Skaftnesmo, Tomasz Furmanek, Frida T. Segafredo, Anders Thorsen, Per Gunnar Fjelldal, Tom Hansen, Geir Lasse Taranger, Eva Andersson, Rüdiger W. Schulz, Anna Wargelius, Rolf B. Edvardsen

**Affiliations:** 1 Institute of Marine Research, P.O. Box 1870, Nordnes, NO-5817 Bergen, Norway; 2 Department of Biology, University of Bergen, Bergen, Norway; 3 Institute of Marine research, Matre Aquaculture Research Station, 5984 Matredal, Norway; 4 Department Biology, Utrecht University, Science Faculty, Padualaan 8, NL-3584 CH Utrecht, The Netherlands

## Abstract

*Vgll3* is linked to age at maturity in Atlantic salmon (*Salmo salar*). However, the molecular mechanisms involving Vgll3 in controlling timing of puberty as well as relevant tissue and cell types are currently unknown. Vgll3 and the associated Hippo pathway has been linked to reduced proliferation activity in different tissues. Analysis of gene expression reveals for the first time that *vgll3* and several members of the Hippo pathway were down-regulated in salmon testis during onset of puberty and remained repressed in maturing testis. In the gonads, we found expression in Sertoli and granulosa cells in males and females, respectively. We hypothesize that *vgll3* negatively regulates Sertoli cell proliferation in testis and therefore acts as an inhibitor of pubertal testis growth. Gonadal expression of *vgll3* is located to somatic cells that are in direct contact with germ cells in both sexes, however our results indicate sex-biased regulation of *vgll3* during puberty.

## Introduction

First discovered in Drosophila, the *vestigial* gene was found to control wing development^1^. Since then, several *vestigial-like* genes have been connected to various cellular processes in vertebrates^2^. Although the roles of the Vestigial-like protein 3 (Vgll3) in vertebrates remain unclear, this cofactor of the Tead family of transcription factors^3^ has been implicated in various processes such as onset of puberty^4,5^, autoimmune diseases^6^, cancer^3^ and fat metabolism^7^. Both Vgll3 and Tead are part of the Hippo signalling pathway, which controls organ size by regulating cell proliferation, differentiation and migration during development of organs^8–10^. The Hippo pathway consists of several proteins that act in a signalling cascade, which negatively regulates the activity of their main target, the cofactors Yap/Taz, ultimately affecting the ability to interact with the partner transcription factor (e.g. Tead). Since the Hippo pathway is a regulator of organ development, we hypothesize that it is involved in pubertal development and growth of the gonads. In fact, the pathway can control testicular and ovarian proliferation in Drosophila^11^, and recent studies have suggested that the Drosophila orthologs Vg (Vgll) and Sd (Tead) together with Tgi (Vgll4) function as default repressors in gonadal escort cells, while Yki (Yap) antagonizes this repression by competing for binding with Sd^12,13^. The pathway has also been linked to ovarian function in human^14^ and to regulating granulosa cell proliferation in the ovary of mice^15^ and chicken^16^. Further, oocytes can stimulate proliferation of granulosa cells in mice by inhibiting the Hippo pathway and increasing the activity of Yap1, while activation of the Hippo pathway promotes granulosa cell differentiation during ovulation^17^. It is known that fat metabolism is related to age at puberty in mice^18^, and interestingly, *Vgll3* has been linked to inhibiting adipocyte differentiation in mouse^7^. Furthermore, a SNP in an enhancer region near VGLL3 in humans was recently linked to reduced body mass index (BMI), body-fat and plasma leptin levels^19^. Very little is known about the role and regulation of *vgll3* in the testis of vertebrates, except for one study showing regulation of *Vgll3* during steroidogenesis in mouse^20^, suggesting a role in testis maturation.

Two recent genome-wide association studies strongly linked *vgll3* to age at maturity in Atlantic salmon (*Salmo salar*)^4,5^. Such a link has been found in human as well, where a single nucleotide polymorphism (SNP) near the *VGLL3* locus has been linked to age at puberty^21^, supporting a conserved but still unknown role for Vgll3 in maturation in vertebrates.

Both wild and farmed Atlantic salmon populations vary greatly in age at sexual maturation^22^. In two recent studies contrasting early and late maturing Atlantic salmon as much as 40% of the age at maturity trait could be explained by SNPs in the *vgll3* locus in chromosome (Chr) 25^4,5^, where two missense mutations were strongly linked to the trait^4^. Environmental cues such as light (photoperiod) and temperature can be used to modulate the timing of male puberty in salmon^22–24^. This is a valuable tool for reducing the otherwise long generation time in salmon, and can shorten the duration of experiments by several years. Taken together with the strong link between *vgll3* and onset of puberty this makes the Atlantic salmon an excellent model to investigate a possible role of Vgll3 in puberty and its connection to the Hippo pathway.

No previous studies have investigated the localization and regulation of *vgll3* during gametogenesis in vertebrates. The aim of this study was therefore to characterize the localization and to start unravelling possible Vgll3 functions in the gonad. In particular, in the testis of a seasonally reproducing species like the Atlantic salmon, the drastic changes in organ size and cell number seem to be candidates for Hippo signalling. To pursue this aim, we used three different experimental setups covering different pubertal stages of Atlantic salmon males. The first experiment included fish just before and just after entering puberty. Fish in the second experiment had progressed further into maturation and contained germ cells in all stages of development, while fish in the third experiment had passed through full maturity and showed testes regressing from the fully mature status. We monitored the expression of *vgll3* and other key players of the Hippo pathway in these experiments. In males, we observed a down-regulation of *vgll3* and Hippo pathway genes during onset of puberty that was maintained during pubertal testis growth, followed by a re-increase in the regressing testis. A down-regulation was not observed in ovaries from an early stage of female puberty. Expression of *vgll3* was localized to Sertoli and granulosa cells, two somatic cell types that directly contact germ cells in males and females, respectively. The results suggest that Vgll3 may be involved in inhibiting Sertoli cell proliferation, thereby connecting the function of Vgll3 to preventing pubertal testis growth in vertebrates.

## Materials and Methods

### Fish samples

All Atlantic salmon (*Salmo salar*) samples used in this study originate from four different experiments and were reared and sampled at Matre Research Station, Matredal, Norway (Table 1). All experiments herein have been approved by the Norwegian Animal Research Authority (NARA); use of the experimental animals was in accordance with the Norwegian Animal Welfare Act of 19th of June 2009.

**Table 1:** Experimental design. Overview of the data used in this study.

**Experiment**	**Strain**	**Type of data**	**Pubertal stages**
Male experiment 1	Aquagen	RNA-Seq, Histology, Sex-steroids, Proliferation	Prepubertal, Early pubertal
Male experiment 2	Mowi	qPCR, Histology, Sex-steroids, Proliferation	Prepubertal, Pubertal
Male experiment 3	Aquagen	qPCR, Histology, Sex-steroids, Proliferation	Prepubertal, Regressing from maturity
Female experiment	Aquagen	qPCR, Histology, Sex-steroids, Proliferation	Prepubertal, Early pubertal

#### Male experiment 1

Testis tissue samples from prepubertal and pubertal salmon males from the Aquagen commercial salmon strain were selected from a group of 2-year-old salmon individually tagged and kept in experimental sea cages (5 × 5 × 8 m, l × w × d). The fish were maintained under ambient light and fed standard commercial diet 3 days a week. Histological analysis, together with gonadosomatic index and plasma androgen levels were used to assess the state of puberty in the males selected. Males just before (prepubertal) and just after (pubertal) entry into puberty were sampled in January and assigned to their groups based on 11-ketotestosterone (11-KT) plasma levels and qualitative histological analysis of proliferation activity. For this experiment the salmon were reared under conditions similar to standard commercial fish farming conditions. Such conditions are listed as an exception in The Norwegian Regulation on Animal Experimentation, thus approval of the experimental protocol of this experiment by NARA was not needed.

#### Male experiment 2

Male fish from a commercial salmon strain (Mowi) were reared according to published protocols for postsmolt maturation induction^24^. Briefly, fish were reared in sea water (16 °C, continuous light) in indoor tanks and fed *ad libitum* with standard commercial diets. After 3,5 months, the fish were anesthetized with Finquel vet and sacrificed by cutting into the *medulla oblongata*. Sex and gonadosomatic index (GSI) were registered and blood samples were taken for hormone measurements. Tissue samples from testis, pituitary, liver and belly flap were collected for gene expression analysis. For this experiment the salmon were reared under conditions similar to standard commercial fish farming conditions. Such conditions are listed as an exception in The Norwegian Regulation on Animal Experimentation, thus approval of the experimental protocol of this experiment by NARA was not needed.

#### Male experiment 3 and Female experiment

Male and female smolts from another commercial salmon strain (Aquagen) were reared in fresh water (16 °C, continuous light) in indoor tanks under conditions which trigger postsmolt maturation^24^. After two months, fish were moved into brackish water (6–12 °C, natural light) for 11 months. The fish were throughout the experimental period fed *ad libitum* on standard commercial diets. Upon sampling, the fish were anesthetized with Finquel vet and sacrificed by cutting into the *medulla oblongata*. Fish sex and GSI were registered and blood samples were taken for hormone measurements. Gonad tissue samples were collected for gene expression analysis. These samples have been previously described in more detail^25^. NARA permit number 5741.

### Histology

Gonad tissue from all fish in Male experiments 2 and 3, and the Female experiment, were fixed in 4% PFA at 4 °C overnight. Subsequently, the tissue was dehydrated, embedded in paraffin, sectioned and then stained with hematoxylin and eosin (HES) according to standard procedures. The sections were inspected with microscopy for scoring of pubertal stages, as detailed for males^23^ and females^26^. In brief, immature testes are characterised by germinal epithelium containing, in addition to Sertoli cells, only type A spermatogonia, while maturing testes contained in addition numerous type B spermatogonia, spermatocytes, spermatids and spermatozoa. Testis regressing from maturity were characterized by tubuli containing a variable number of spermatozoa, reflecting different degrees of phagocytotic removal of unused spermatozoa by Sertoli cells, while the germinal epithelium started to become re-established and contained a layer of Sertoli cells and type A spermatogonia, in particular in areas where sperm resorption had progressed considerably. Immature and early pubertal testes were distinguished by assessment of mitotic activity, which can be observed in the nuclei of type A spermatogonia and Sertoli cells in the early pubertal testes. Cell proliferation was assessed by immunocytochemical localization of the proliferation marker phosphorylated histone H3 (pH3)^27,28^ in male and female gonads. Two sections of 5 µm that were at least 100 µm apart from each other were used for detection of pH3 as described elsewhere^29^, except that the primary antibody was detected by undiluted HRP-conjugated goat antirabbit IgG (Brightvision Immunologic, AH Diagnostics) for 30 min. This proliferation analysis has already been carried out and reported for the differentiation between immature and early pubertal testis samples^30^. Histological slides from three maturing and regressing testes, and prepubertal and early pubertal ovaries, were scanned with a digital slide scanner (Hamamatsu NanoZoomer S60) using a 40× source lens (resolution 220 nm/pixel). From each of the digital slides, 10 non-overlapping subareas of 280 × 280 µm were selected randomly and exported at full resolution for quantification of the pH3-positive area fractions. This was achieved using the open source image analyser program ImageJ (https://imagej.nih.gov/ij/), the ObjectJ plugin (https://sils.fnwi.uva.nl/bcb/objectj/), and the Weibel grid project file (https://sils.fnwi.uva.nl/bcb/objectj/examples/Weibel/MD/weibel.html). The counting grid employed was a Weibel grid^31^ with 297 grid lines (594 grid points) for each sub-area. Two-way ANOVA in Prism 7 (GraphPad) was used to compare the stained area fractions between the different groups.

### Steroid hormone quantification

Analysis of sex steroids was performed for 11-KT and estradiol-17β (E2) using ELISA^32^ and validated as previously described^26^.

### Testis RNA-seq analysis

From Male experiment 1, total RNA was extracted from testis samples in three biological replicates per pubertal stage using miRNeasy Mini Kit (Qiagen) and treated with TURBO DNase-Free kit (Ambion). RNA quality was inspected using Nanodrop (all samples had 260/280-ratio ≥ 1.9) and RNA integrity was checked using BioAnalyzer (Agilent) (all samples had RIN ≥ 8). RNA was sequenced on the HiSeq.2000 sequencing platform (Illumina), and made available on SRA with BioProject ID PRJNA380580. Sequenced reads were mapped to the reference gene models for Atlantic salmon (NCBI *Salmo salar* Annotation Release 100) using Bowtie2^33^. Read counts were summed for each unique GeneID and normalized by total mapped read counts. Statistical analysis for detection of differentially expressed genes was done using NOISeqBIO from the NOISeq package^34^, with threshold of P_NOI_ = 0.95, corresponding to false discovery rate adjusted P-values of 0.05. KEGG pathway analysis^35^ was performed by mapping the KEGG annotated differentially expressed genes (DEGs) from NOISeqBIO to KEGG. Regulation of pathways between different stages was investigated by comparing the proportion of up-regulated DEGs to down-regulated DEGs in each pathway, only including significant DEGs (P < 0.05, fold change > 1.5) and pathways with ≥10 DEGs, and excluding pathways listed as Human Diseases. To obtain tissue specific expression profiles, RNA-seq reads from a variety of salmon tissue were downloaded from SRA (BioProject ID PRJNA72713) and mapped with Bowtie2 and normalized by total read counts. Expression of KEGG Hippo pathway genes in testis and selected Hippo genes in other tissues were illustrated with heatmaps made with J-Express v. 2012^36^, using high level mean and variance normalization, and clustered with complete linkage and Euclidean distance measure.

### qPCR

All RNA isolation prior to qPCR was done using iPrep Total RNA Kit (Invitrogen), except for belly flap where RNA was isolated using miRNeasy Mini Kit (Qiagen). The RNA was treated with TURBO DNAse (Ambion), followed by treatment with DNase Inactivation Reagent (Ambion). RNA integrity was inspected on a subset of the samples using BioAnalyzer (Agilent). cDNA was synthesized using VILO SuperScript cDNA Synthesis Kit (Invitrogen), using equal amounts of RNA in each reaction. qPCR was performed in duplicates with gene-specific primers designed to avoid amplification of paralogs, listed in Supplementary Table S1, using PowerUp SYBR Green Master Mix (Applied Biosystems) on the QuantStudio 5 Real-Time PCR System (ThermoFisher). Elongation factor 1 alpha (*elf1a*) was used as endogenous control. It is preferable to include more than one single endogenous control, therefore we also used Secretory Carrier Membrane Protein 1 (*scamp1*) which is involved in post-golgi recycling pathways. This was found to be one of the most stably expressed genes during male puberty in the RNA-seq data, with normalized read counts ranging from 1,681–1,873 (SD:61.3). Standard curves were made for each gene using 4-fold dilution series (Supplementary Table S1). Melt curves were inspected to ensure amplification of single amplicons. Statistical analysis was done with the ΔΔCt approach, using the geometric mean of both endogenous controls for calculating ΔCt, and calibrating the measurements so that the mean fold change of the prepubertal groups becomes 1. Significance testing was done in Prism 7 (GraphPad) using the nonparametric Mann-Whitney test, since D’Agostino & Pearson normality test showed that some of the data was not normally distributed.

### **cRNA probe synthesis and*in situ*hybridization**

PCR was done with *vgll3*-specific primers with added Sp6/T7 sequences, and cRNA probes for *in situ* hybridization were produced from gDNA with DIG-AP RNA Labeling Kit (Sp6/T7, Roche Diagnostics). Primers used for generating the probe for *vgll3* are listed in Supplementary Table S2. Probes were precipitated, washed and resuspended as previously described^37^. Antisense and sense were produced by T7 and Sp6 polymerases, respectively. Quality of the probes was inspected with BioAnalyzer (Agilent), and DIG incorporation estimated with a spot-test. *In situ* hybridization with digoxigenin-alkaline phosphatase (DIG-AP), including preparation of tissue, was done on cryosections as previously described^37^ on prepubertal testis and ovary, and from germ cell-free testis (described elsewhere^25,38^).

### **Bioinformatical analysis of*vgll3*paralogs**

Six copies of *vgll3* are found in the Atlantic salmon reference genome^39^. We believe only two of these are real, the others being artefacts from the genome assembly process. Sequence similarities among all copies were investigated by multiple sequence alignment using Muscle^40^. On Chr 21 two copies of *vgll3* are contained within a tandemly duplicated region. To show that this tandem duplication is incorrect, and that only a single *vgll3* exists in Chr 21, we inspected depth of coverage in genomic sequences from two different Atlantic salmon populations (BioProject ID PRJNA293012, Suldalslågen and Eidselva). Genomic sequences were mapped to the salmon reference genome using Bowtie2, and sequences mapping to Chr 21 were remapped to a new version of the chromosome where the second duplicate has been removed (replaced with Ns). Depth of coverage for properly paired reads, with and without the second duplicated region, was found using Samtools depth^41^. To show the sequence similarity of the duplicated regions, sequences in 10 kb genomic windows spanning both duplicates were aligned against each other using BLASTN^42^.

### Data Availability

Transcriptome sequence data used in this study have been made available on SRA with Bioproject number PRJNA380580.

## Results and Discussion

**Figure 1:**
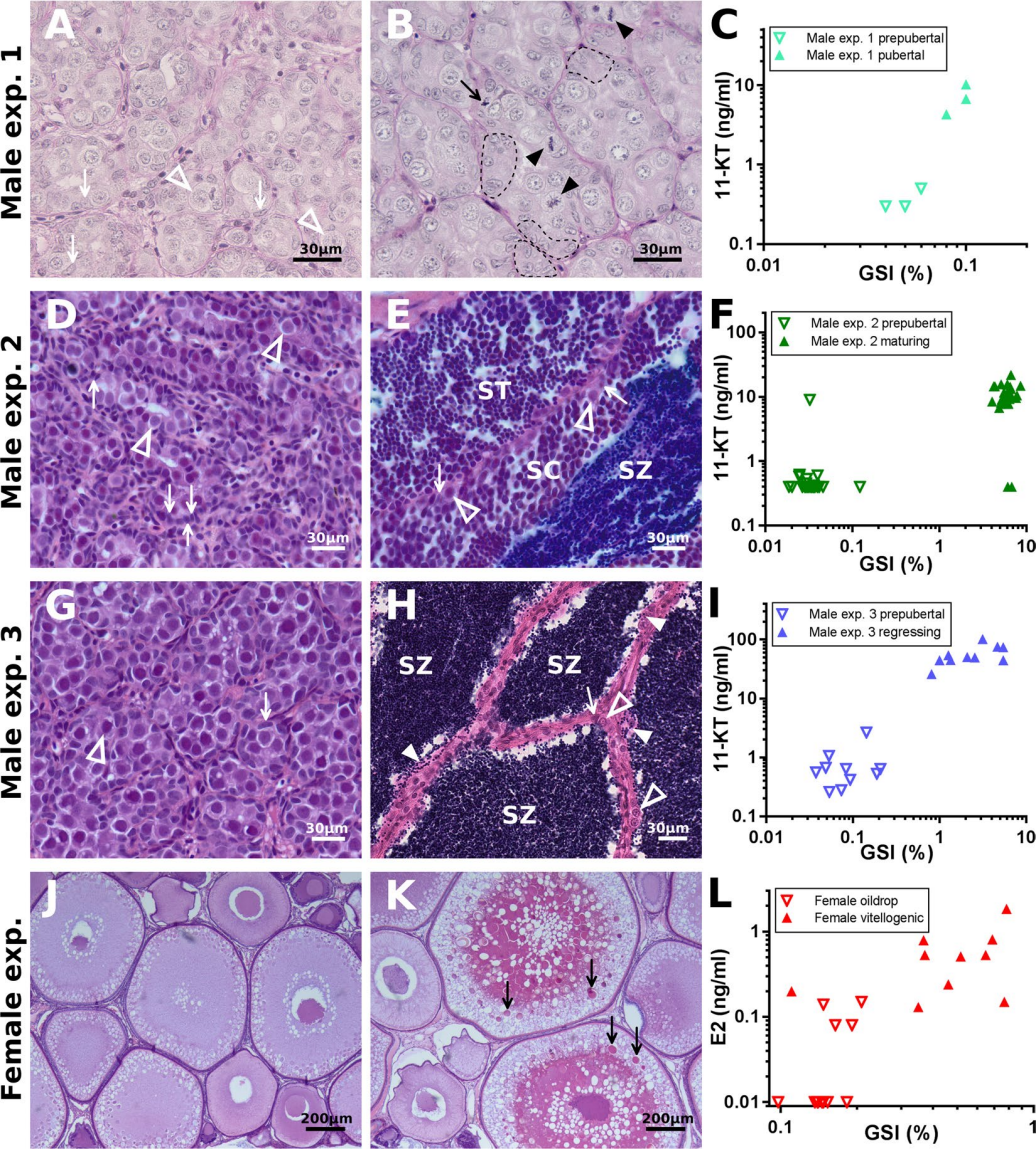
Characterization of pubertal stages. Histological illustrations of gonads from the four different experiments used in this study, showing (**A)** prepubertal and (**B)** pubertal testis from Male experiment 1 with (**C**) GSI and 11-ketotestosterone (11-KT), (**D)** prepubertal and (**E)** maturing testis from Male experiment 2 with (**F)** GSI and 11-KT, and (**G)** prepubertal and (**H)** testis regressing from maturity from Male experiment 3 with (**I)** GSI and 11-KT, and finally (**J)** oildrop (prepubertal) and (**K)** early vitellogenic (early puberty) ovary from the Female experiment with (**L)** GSI and estradiol-17β (E2). The scale bar is 30 µm in all pictures, except in (**J** and **K)**, which are 200 µm. In (**B**), elevated mitotic activity is shown in type A spermatogonia (black arrowheads) and Sertoli cells (black arrow), and stippled lines show accumulations of Sertoli cells not yet in contact with germ cells. In (**A)** and (**D**–**H)**, open white arrowheads indicate type A spermatogonia, white arrows indicate Sertoli cells. In (**E** and **H)**, two letter codes indicate spermatogenic cysts containing spermatocytes (SC) or spermatids (ST); spermatozoa in the lumen of spermatogenic tubules are labelled by SZ. In (**H)**, white arrowheads indicate removal of sperm by Sertoli cells. In (**K)**, the yolk vesicles containing vitellogenin are indicated by black arrows.

Farmed Atlantic salmon from three male experiments and one female experiment, covering four different pubertal stages in males and two in females, were used in this study. Male experiment 1 covered prepubertal and pubertal stages, providing good resolution around the onset of puberty, with staging based on plasma androgen levels, GSI and histology (Fig. 1A–C). Male experiment 2 contained prepubertal and maturing fish (Fig. 1D–F), while Male experiment 3 included prepubertal fish and fish regressing from maturity (Fig. 1G–I). For the male fish, pubertal stages were determined using 11-ketotestosterone (11-KT), GSI and histology. To provide quantitative measures for proliferation and degree of immaturity in testis samples, *pcna*^43,44^ and *amh*^45^ expression were measured, respectively (Supplementary Fig. S1). *pcna* was significantly up-regulated in testes from fish just entering maturity and in pubertal fish in comparison to prepubertal fish. We have previously published observations that early pubertal testes has a higher Sertoli cell proliferation activity compared to prepubertal testes when assayed using immunocytochemical detection of pH3^30^. Similarly, when quantifying the number of pH3-positive cells in prepubertal and early pubertal testis samples, we have found a clear, maturation-associated increase in the number of proliferating somatic and germ cells^23^. To assay if proliferation activity could also be recorded in maturing testes, we carried out pH3 immunodetection also on testis sections from these fish. We found that both germ and Sertoli cell proliferation can be observed in these samples (Supplementary Fig. S2), and that proliferation activity was much higher in maturing than in regressing testes. As expected, *pcna* transcript levels were not different in testis regressing from maturity, since germ and somatic cell proliferation had been completed for the present reproductive cycle in these testes. This was supported by the observation that pH3-positive cells were rarely found in the testes regressing from full maturity (Supplementary Fig. S2). Female pubertal stages were determined from estradiol-17β (E2), GSI and histology (Fig. 1J–L). These measurements were well-suited for pinpointing the pubertal status in salmon^26,45^ and allowed clear separation of fish in different pubertal stages. Expression of *pcna* was measured in ovaries from immature and early vitellogenic stages, but no significant differences were detected (Supplementary Fig. S1). Also, the morphological proliferation studies showed no differences in proliferation activity among somatic cells in the ovaries comparing prepubertal and early pubertal ovaries; some isolated, proliferating granulosa cells were found in some of the follicles but there were no clear differences visible in females, in stark contrast to somatic and germ cell proliferation activity in males in different stages of puberty (Supplementary Fig. S2).

**Figure 2:**
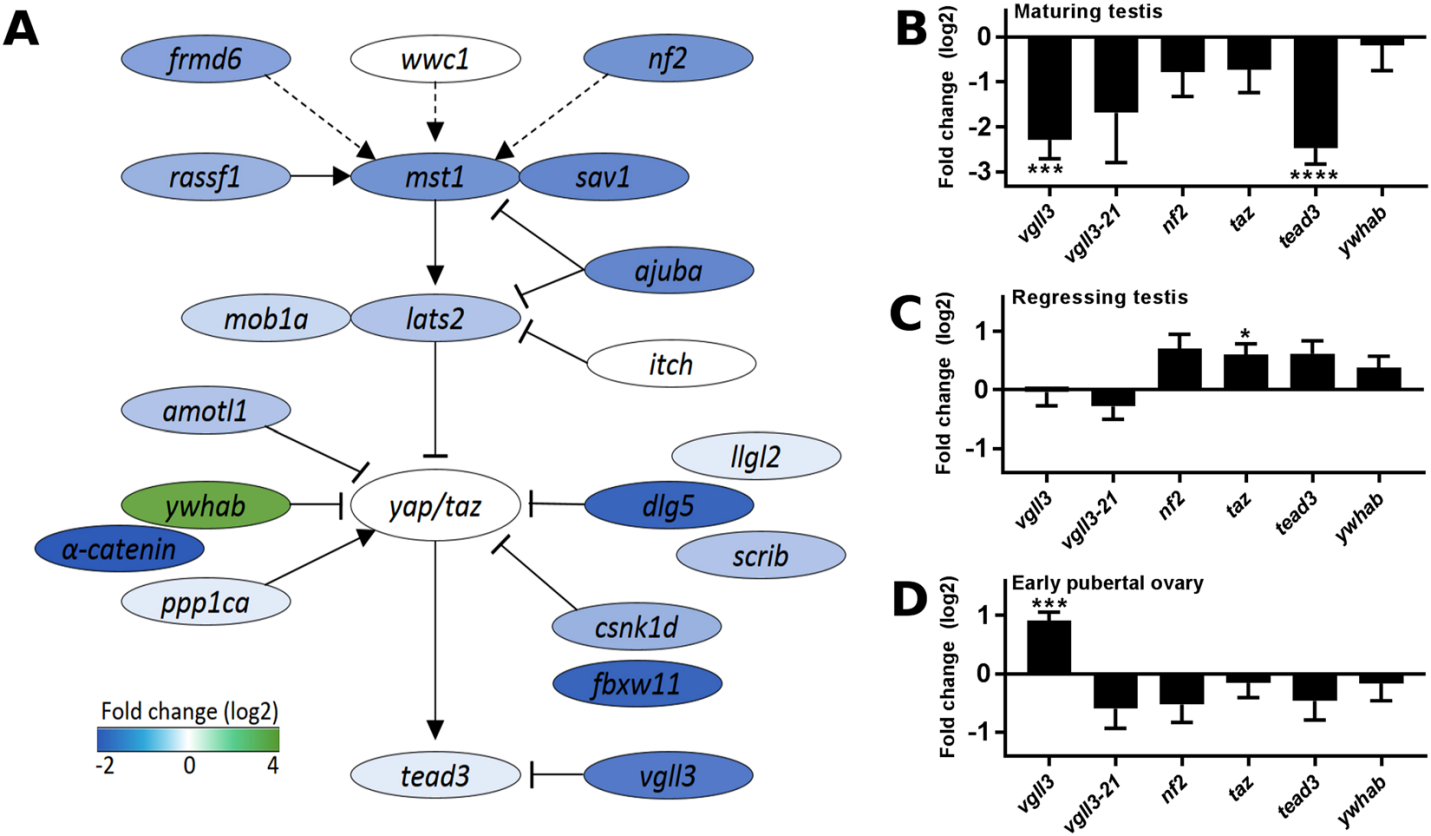
Regulation of the Hippo pathway in salmon maturation. (**A)** Regulation of the Hippo pathway genes (gene names shown in Supplementary Fig. S5) in testis from prepubertal to pubertal stages, inferred by RNA-seq with Male experiment 1. Green and blue indicate up and down-regulation, respectively, and white colour means not regulated. Fold change of gene expression for selected Hippo genes measured by qPCR for (**B)** prepubertal to maturing testis from Male experiment 2 (n = 15 in each group, and n = 8 for *vgll3*_21_ in the maturing group), (**C)** prepubertal to testis regressing from maturity from Male experiment 3 (n = 8–10 in each group), and (**D)** from oildrop (prepubertal) to early vitellogenic (early puberty) stages in ovaries from the Female experiment (n = 10–13 in each group). Error bars show SEM. *P < 0.05, **P < 0.01, ***P < 0.001, ****P < 0.0001.

Gene expression in testis from 3 prepubertal and 3 pubertal males (Male experiment 1), were studied by RNA-seq. Mapping sequence data to the reference gene models of Atlantic salmon revealed a significant (P < 0.05, fold change > 1.5) down-regulation of 16,857 genes and up-regulation of 4,464 genes from the prepubertal to the pubertal stage. To highlight the most important differences we examined which KEGG pathways were differentially regulated by comparing the ratio of up and down-regulated genes in each pathway (Supplementary Fig. S3). “DNA replication” and “Oxidative phosphorylation” were among the most up-regulated pathways in testis, indicating a change in metabolic rate at this stage. The most down-regulated pathways included “Signaling pathways regulating pluripotency of stem cells” and “Hippo signaling pathway” (Supplementary Fig. S3). Interestingly, we discovered that *vgll3* and several of the genes in the Hippo pathway, such as *mst1*, *nf2* and *tead3* were significantly (P < 0.05) down-regulated in the pubertal compared to the prepubertal testis (Fig. 2A and Supplementary Fig. S3, Supplementary Dataset S1). To investigate the regulation of the Hippo pathway at other maturation stages, we selected a set of important Hippo genes^9,10,46^ based on level of expression and differential significance (P ≤ 5%) in testis RNA-seq data, and included *taz, vgll3* and *tead3*, a factor recently shown to influence early maturation in salmon^47^. RNA-seq results showed that both *vgll3* and the other selected Hippo genes were significantly down-regulated from the prepubertal stage to the pubertal stage (Fig. 2A). The only up-regulated gene in Fig. 2A was *ywhab*, which is known to promote cytoplasmic retention of Yap/Taz^48^, and interestingly, *ywha* genes accounted for 37% (n = 14) of the significantly up-regulated genes in the pubertal stage (Supplementary Fig. S3). Figure 2A illustrates that both *vgll3* and the Hippo pathway are to a large extent down-regulated in testis during puberty, with only a few exceptions.

To confirm the regulation of *vgll3* and the Hippo pathway observed by RNA-seq and to include later pubertal stages, we examined gene expression of six selected Hippo genes, including *vgll3*, using qPCR. Here we used testis from prepubertal, maturing and testis regressing from maturity (Male experiments 2 and 3). This confirmed the down-regulation of *vgll3* and the Hippo pathway during puberty (Fig. 2B), and revealed a subsequent up-regulation when the fish were regressing from the mature status (Fig. 2C). We did not observe the up-regulation of *ywhab* shown by RNA-seq.

**Figure 3:**
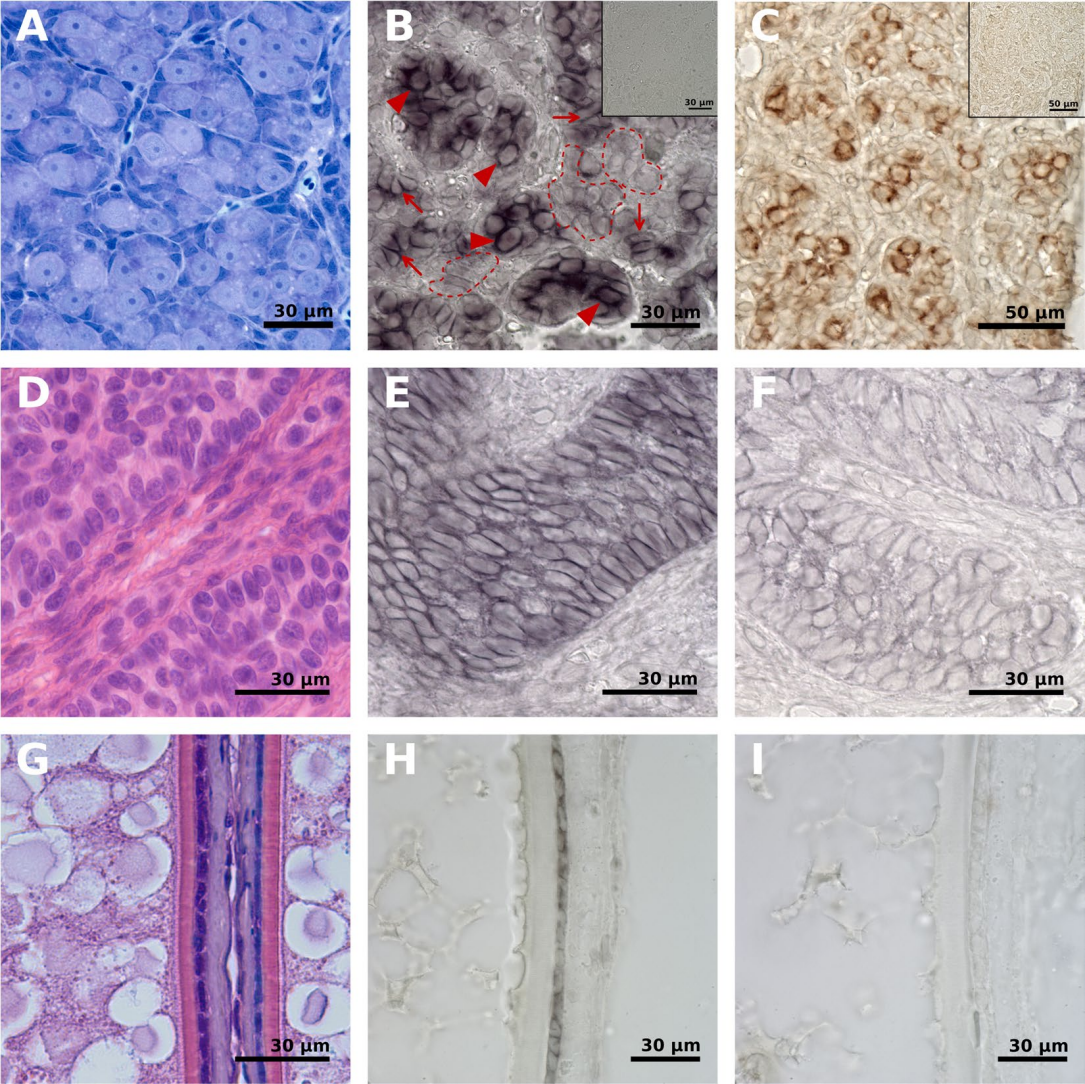
*In situ* hybridization shows localization of *vgll3* in gonads. (**A)** HES staining tissue sections of prepubertal testis, (**B)** expression of *vgll3* in Sertoli cells in prepubertal testis, with negative control shown in the upper right corner. (**C)** The germ cell specific gene *piwi* used as a germ cell marker by *in situ* hybridization on an immature testis, with negative control shown in the upper right corner. (**D)** HES staining tissue sections of germ cell free testis, (**E**) expression of *vgll3* in germ cell free testis, showing expression in Sertoli cells, and (**F**) negative control. (**G)** HES staining tissue sections of vitellogenic ovary, (**H)** expression of *vgll3* in granulosa cells in vitellogenic ovary, and (**I)** negative control. The scale bars in (**A**,**B**,**D**–**I**) is 30 µm, and 50 µm in (**C**). Annotations show areas with Sertoli cells in contact with type A spermatogonia (arrowheads) and other Sertoli cells (arrows), and areas within spermatogenic tubules with little or no labelling (stippled lines).

*In situ* hybridization served to identify the cell types in salmon testis expressing *vgll3*. Using sections from immature testis (expressing high levels of *vgll3*; see Fig. 2A), we observed expression in Sertoli cell cytoplasm, showing for the first time that *vgll3* is mainly expressed in this cell type in the testis (Fig. 3A–C). It should be noted that at this point we cannot distinguish *vgll3* paralogs with the *in situ* method due to the high similarity in sequence. However, specific expression of the *vgll3* paralogs were restricted to Sertoli cells. To further ascertain the expression of *vgll3* in Sertoli cells, we used a germ cell free testis model, produced by knocking out the *dnd* gene^38^. In these fish, the testicular tubules contain only Sertoli cells and we observed clear expression of *vgll3* in Sertoli cell clusters found in germ cell-free testis (Fig. 3D–F). We also performed an *in situ* hybridization assay using the germ cell specific marker *piwi*^49^, and our data shows that *vgll3* is not found in the same cell types as *piwi* (Fig. 3C). Sertoli cells are the only somatic cell type in the spermatogenic tubule and provide germ cells with physical, metabolic and regulatory support; during all stages of spermatogenesis, germ cells depend on this support by Sertoli cells^50^. In fish, Sertoli cells form intratubular spermatogenic cysts. Starting with one or two Sertoli cells that envelope a single, undifferentiated type A spermatogonium, this germ cell proceeds through a genetically fixed number of mitotic divisions before completing the two meiotic cell cycles and spermiogenesis. Hence, inside a spermatogenic cyst, a single germ cell clone proceeds in synchrony through the different stages of spermatogenesis. Since the germ cell number duplicates with each cell cycle, also the cyst-forming Sertoli cells need to proliferate to provide additional space for the growing germ cell clone. Another type of Sertoli cell proliferation occurs in context with the production of new spermatogenic cysts. In Atlantic salmon entering puberty, this leads to a situation, in which Sertoli cell numbers increase clearly, such that groups of Sertoli cells appear that are not (yet) in contact with germ cells (Fig. 1B). When single type A spermatogonia become available, they can associate with “idle” Sertoli cells and form new spermatogenic cysts^51^. While the cellular composition within the spermatogenic tubules is similar in prepubertal and pubertal stages in experiment 1 (Sertoli cells and type A spermatogonia), the levels of testicular activity were clearly different (Fig. 1A–C). Within the spermatogenic tubules, the increased level of activity is indicated by the elevated mitotic activity among type A spermatogonia (Fig. 1B, arrowheads) and Sertoli cells (Fig. 1B, arrow); in some areas (Fig. 1B, stippled lines), Sertoli cells have accumulated and form somatic cell groups not (yet) contacting germ cells. Quantification of markers for immature testis (*amh*) and cell proliferation (*pcna*) confirm these changes as *amh* is down-regulated and *pcna* is up-regulated in early pubertal testis relative to prepubertal testis (Supplementary Fig. S1). Closer inspection of the *vgll3 in situ* hybridization staining pattern showed that the label is concentrated in areas where Sertoli cells contact type A spermatogonia (Fig. 3B, arrowheads), whereas in areas where two or more Sertoli cells contact each other, the label was less intense and evenly distributed (Fig. 3B, arrows). This observation suggests that germ-Sertoli cell interaction can modulate the intracellular location and/or post-transcriptional use of the *vgll3* transcript. Finally, some areas within the spermatogenic tubules showed little or no labelling (Fig. 3B, stippled lines). We also examined *vgll3* expression in germ cell-free testis^38^. We observed a clear staining signal for *vgll3* in Sertoli cell cytoplasm (Fig. 3D–F), and interestingly, here we did not observe any discrete cytoplasmic localization of *vgll3*.

While Yap/Taz promote proliferation, the Hippo pathway counteracts this by inhibiting Yap/Taz activity and preventing its interaction with Tead^9^. Considering the down-regulation of *vgll3* and Hippo pathway members in pubertal testis, and the report that the Hippo pathway gene *expanded* has been found to be involved in control of spermatogonial proliferation in Drosophila^11^, we hypothesize that Vgll3 and other players in the Hippo pathway control the proliferation of Sertoli cells during the onset of puberty. As discussed above, Sertoli cell proliferation either leads to the production of new spermatogenic cysts, or facilitates the growth of an existing cyst. In line with our hypothesis that Vgll3 hinders proliferation, we interpret intratubular areas showing low or no *vgll3* signal as areas where Sertoli cells are more likely to proliferate, preparing the ground for an expansion of the population of early spermatogonia (production of new cysts; Figs 2A and 3B). Later on, in maturing testes the still low *vgll3* transcript levels (Fig. 2B) may be required to allow the high proliferation activity in maturing testes (Supplementary Fig. S2) that is only possible when the associated Sertoli cells proliferate as well in order to generate the space required for the growing germ cell clones^52^. Finally, the re-increase of *vgll3* transcript levels in testes regressing from maturity can be understood in the context of proliferation as well. In these regressing testes, the only germ cell type next to spermatozoa are type A undifferentiated spermatogonia^53^ that are in contact with Sertoli cells. They form the basis for next year’s spermatogenic wave and are part of the subsequent reproductive cycle. It is therefore not surprising that the proliferation activity was very low in these regressing testes (Supplementary Fig. S2), associated with the presence of re-elevated *vgll3* levels. Accordingly, the proliferation marker *pcna* was not up-regulated in the regressing testes, in contrast to early pubertal and pubertal testes, relative to prepubertal testes (Supplementary Fig. S1).

We also investigated whether *vgll3* and the Hippo pathway are regulated during female puberty. Expressional differences of *vgll3* and a set of other Hippo genes between ovaries from oildrop (prepubertal) and early vitellogenic (early puberty) females was examined by qPCR. None of the Hippo genes were differentially expressed between the two stages, with a striking exception of *vgll3*, which was up-regulated in the fish that had entered early vitellogenesis (Fig. 2D). The ovary samples from the female experiment only included early stages of vitellogenesis. Therefore, gene expression was also assayed in samples from ovaries in late vitellogenesis previously described by and obtained from Andersson *et al*.^26^. No significant regulation was detected in any of the Hippo genes. *vgll3* showed a trend of being up-regulated, however this was not significant (P = 0.0519, Supplementary Fig. S4). Further, from available Atlantic salmon tissue RNA-seq data we observed that some Hippo genes are mostly expressed in ovaries, while others are more expressed in testis (Supplementary Fig. S5).

By *in situ* hybridization in an early vitellogenic ovary, *vgll3* was found to be expressed in granulosa cells (Fig. 3G–I), the somatic cell type in the ovary being in direct contact with the germ cells and fulfilling functions equivalent to those of Sertoli cells in the testis^54^. In mice, down-regulation of the Hippo pathway before ovulation, induced granulosa cell proliferation^17^. Furthermore, by knock-down of the Hippo component SAV1, granulosa cell proliferation in chicken was de-inhibited^16^. In salmon, the Hippo genes were not regulated between the oildrop (prepubertal) stage and any of the vitellogenic stages, but *vgll3* was up-regulated in the early vitellogenic stage (Fig. 2D, Supplementary Fig. S4). This could indicate that granulosa cell proliferation is limited at the vitellogenic stages. The samples from the female experiment were derived from an experiment where photoperiod and temperature conditions stimulated postsmolt maturation. Usually, only males complete maturation under these conditions since at this age and size, the body mass of females is too small to support full maturation. Moreover, also in older/larger females having reached the early vitellogenic stage, the continuation from early into full vitellogenesis can be interrupted, under certain photoperiod conditions^26^. This could indicate that onset of puberty is regulated differently in females and males, which may be related to the finding that genotypes of salmon *vgll3* have different effects on the age at maturity in males and females^5^. This data was supported by *pcna* expression, where early vitellogenic ovaries did neither show elevated levels of the proliferation marker *pcna*, nor differences in the proliferation activity as assayed immunocytochemically, both in contrast to what was observed for testis (Supplementary Figs S1 and S2). Future studies will reveal how regulation of *vgll3* and the Hippo pathway affect proliferation and differentiation of Sertoli cells and if granulosa cell proliferation responds to this signalling system as well.

Salmon has gone through a whole genome duplication ~80 million years ago, resulting in a genome where 55% of the genes are duplicated^39^. The Atlantic salmon genome contains two copies of *vgll3*, one on chromosome 25 which has been linked to age at maturity, and a paralog on chromosome 21 referred to as *vgll3*_21_ in this study. The two copies share 90% sequence similarity, but it is not known if they share function. Since *vgll3* on Chr 25 has been linked to age at maturity this study focus mostly on this copy, but it is also relevant to investigate if and how *vgll3*_21_ is regulated during puberty. This also enables us to check that we are amplifying the correct *vgll3* transcript in the qPCR analysis. Multiple copies of *vgll3*_21_ are present in the reference genome assembly, but analysing sequence similarity and mapping depth of resequencing data, we found that the multiple copies are most likely artefacts created during genome assembly (Supplementary Fig. S6). We conclude that the genome contains only two *vgll3* genes. By RNA-seq we observed that *vgll3*_21_ was also differentially expressed in testis, though with a lower fold change (1.88 for *vgll3* vs. 1.48 for *vgll3*_21_). However, qPCR showed no significant regulation of *vgll3*_21_ in male experiment 2 and 3, and in females (Fig. 2B–D).

**Figure 4:**
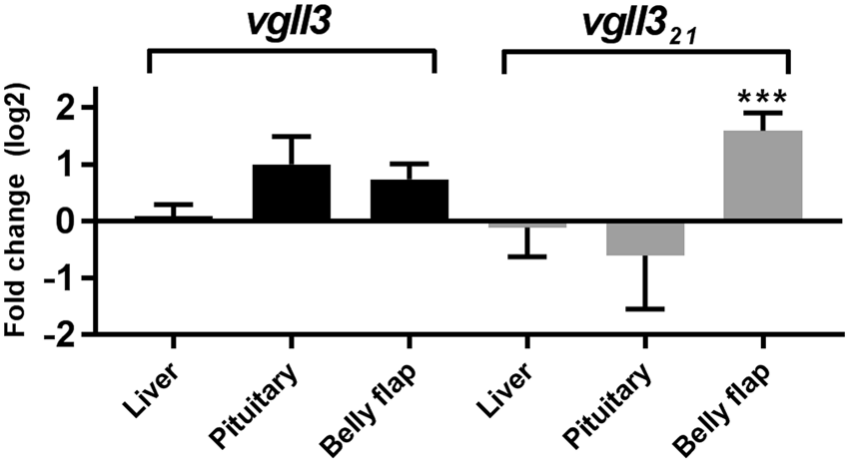
Expression of *vgll3* in liver, pituitary and belly flap. Fold change of *vgll3* and *vgll3*_21_ gene expression from prepubertal to maturing stages in liver, pituitary and belly flap from Male experiment 2 (n = 10–12 for liver and belly flap in both stages, and n = 6–8 for pituitary in both stages). ***P < 0.001.

In mouse, *vgll3* has been linked to inhibition of adipocyte differentiation^7^, and a SNP in an enhancer region near VGLL3 has been linked to reduced body mass index, body-fat and leptin levels in human^19^, prompting us to investigate if the gene is regulated in salmon adipose tissue during puberty. Belly flap contains much adipose tissue, and increased adiposity has been associated with maturation in salmon^55–58^. We therefore examined the expression of *vgll3* in this tissue, and in liver and pituitary, from fish in Male experiment 2 (Fig. 4). No significant differences were found in pituitary and liver, but in belly flap *vgll3*_21_ was significantly up-regulated, correlating with the reports in mouse^7^ and the reported link between fat reserves and maturation in salmon. However, this regulation was not observed for the Chr 25 paralog which is the one linked to maturation^4^. The tissue expression profiles show that the two *vgll3* paralogs are expressed in the same tissues and at similar levels, with gill, heart and testis showing the highest expression (Supplementary Fig. S5). Still, one could speculate that the *vgll3* paralogs have sub-functionalized in salmon, which has given the paralogs different functions in regulation of puberty.

## Conclusion

Our results demonstrate that *vgll3* and the Hippo pathway are down-regulated in testis during the entry into puberty in Atlantic salmon, followed by an up-regulation in testis regressing from maturity. In context with the strong genetic link to age at maturity in salmon and studies describing the Hippo pathway as a controller of proliferation and organ size in other animals, we propose that *vgll3* has a role in restricting pubertal testis growth and the associated spermatogenic development in Atlantic salmon males. The fact that *vgll3* and the Hippo pathway are conserved in vertebrates and have been linked to pubertal functions in other species suggests a conserved mechanism where Vgll3 is part of a network of proteins regulating pubertal growth of the gonads in vertebrates. Importantly, this study also shows for the first time that *vgll3* is expressed in Sertoli and granulosa cells. Finally, our data show a difference in the regulation of *vgll3* transcript levels between testis and ovary in salmon entering puberty, indicating that this gene plays different roles in the two sexes.

## Electronic supplementary material

Supplementary information

Supplementary Dataset S1

## References

[1] Williams JA, Bell JB, Carroll SB (1991). Control of Drosophila wing and haltere development by the nuclear vestigial gene product. Genes Dev.

[2] Simon E, Faucheux C, Zider A, Theze N, Thiebaud P (2016). From vestigial to vestigial-like: the Drosophila gene that has taken wing. Dev Genes Evol.

[3] Helias-Rodzewicz Z (2010). YAP1 and VGLL3, encoding two cofactors of TEAD transcription factors, are amplified and overexpressed in a subset of soft tissue sarcomas. Genes Chromosomes Cancer.

[4] Ayllon F (2015). The vgll3 Locus Controls Age at Maturity in Wild and Domesticated Atlantic Salmon (Salmo salar L.) Males. PLoS Genet.

[5] Barson NJ (2015). Sex-dependent dominance at a single locus maintains variation in age at maturity in salmon. Nature.

[6] Liang Y (2017). A gene network regulated by the transcription factor VGLL3 as a promoter of sex-biased autoimmune diseases. Nat Immunol.

[7] Halperin DS, Pan C, Lusis AJ, Tontonoz P (2013). Vestigial-like 3 is an inhibitor of adipocyte differentiation. J Lipid Res.

[8] Huang J, Wu S, Barrera J, Matthews K, Pan D (2005). The Hippo signaling pathway coordinately regulates cell proliferation and apoptosis by inactivating Yorkie, the Drosophila Homolog of YAP. Cell.

[9] Meng Z, Moroishi T, Guan KL (2016). Mechanisms of Hippo pathway regulation. Genes Dev.

[10] Plouffe SW, Hong AW, Guan KL (2015). Disease implications of the Hippo/YAP pathway. Trends Mol Med.

[11] Sun S, Zhao S, Wang Z (2008). Genes of Hippo signaling network act unconventionally in the control of germline proliferation in Drosophila. Dev Dyn.

[12] Koontz LM (2013). The Hippo effector Yorkie controls normal tissue growth by antagonizing scalloped-mediated default repression. Dev Cell.

[13] Li C (2015). Ci antagonizes Hippo signaling in the somatic cells of the ovary to drive germline stem cell differentiation. Cell Res.

[14] Kawamura K (2013). Hippo signaling disruption and Akt stimulation of ovarian follicles for infertility treatment. Proc Natl Acad Sci USA.

[15] Xiang C (2015). Hippo signaling pathway reveals a spatio-temporal correlation with the size of primordial follicle pool in mice. Cell Physiol Biochem.

[16] Lyu Z (2016). The Hippo/MST Pathway Member SAV1 Plays a Suppressive Role in Development of the Prehierarchical Follicles in Hen Ovary. PLoS One.

[17] Sun, T. *The roles of Hippo signaling pathway in mouse ovarian function*, The Pennsylvania State University (2016).

[18] Ahima RS, Dushay J, Flier SN, Prabakaran D, Flier JS (1997). Leptin accelerates the onset of puberty in normal female mice. J Clin Invest.

[19] Nakayama, K., Ohashi, J., Watanabe, K., Munkhtulga, L. & Iwamoto, S. Evidence for Very Recent Positive Selection in Mongolians. *Mol Biol Evol*, 10.1093/molbev/msx138 (2017). 10.1093/molbev/msx138 28444381

[20] McDowell, E. N. *et al*. A Transcriptome-Wide Screen for mRNAs Enriched in Fetal Leydig Cells: CRHR1 Agonism Stimulates Rat and Mouse Fetal Testis Steroidogenesis. *Plos One* **7**, 10.1371/journal.pone.0047359 (2012). 10.1371/journal.pone.0047359 PMC3484991 23133512

[21] Cousminer DL (2013). Genome-wide association and longitudinal analyses reveal genetic loci linking pubertal height growth, pubertal timing and childhood adiposity. Hum Mol Genet.

[22] Taranger GL (2010). Control of puberty in farmed fish. Gen Comp Endocrinol.

[23] Melo MC (2014). Salinity and photoperiod modulate pubertal development in Atlantic salmon (Salmo salar). J Endocrinol.

[24] Fjelldal PG, Hansen T, Huang TS (2011). Continuous light and elevated temperature can trigger maturation both during and immediately after smoltification in male Atlantic salmon (Salmo salar). Aquaculture.

[25] Kleppe L (2017). Sex steroid production associated with puberty is absent in germ cell-free salmon. Scientific Reports.

[26] Andersson E (2013). Pituitary gonadotropin and ovarian gonadotropin receptor transcript levels: seasonal and photoperiod-induced changes in the reproductive physiology of female Atlantic salmon (Salmo salar). Gen Comp Endocrinol.

[27] Cobb J, Miyaike M, Kikuchi A, Handel MA (1999). Meiotic events at the centromeric heterochromatin: histone H3 phosphorylation, topoisomerase II alpha localization and chromosome condensation. Chromosoma.

[28] Hendzel MJ (1997). Mitosis-specific phosphorylation of histone H3 initiates primarily within pericentromeric heterochromatin during G2 and spreads in an ordered fashion coincident with mitotic chromosome condensation. Chromosoma.

[29] Almeida FF, Kristoffersen C, Taranger GL, Schulz RW (2008). Spermatogenesis in Atlantic cod (Gadus morhua): a novel model of cystic germ cell development. Biol Reprod.

[30] Skaftnesmo KO (2017). Integrative testis transcriptome analysis reveals differentially expressed miRNAs and their mRNA targets during early puberty in Atlantic salmon. BMC Genomics.

[31] Weibel ER, Kistler GS, Scherle WF (1966). Practical stereological methods for morphometric cytology. J Cell Biol.

[32] Cuisset B (1994). Enzyme-Immunoassay for 11-Ketotestosterone Using Acetylcholinesterase as Label - Application to the Measurement of 11-Ketotestosterone in Plasma of Siberian Sturgeon. Comp Biochem Phys C.

[33] Langmead B, Salzberg SL (2012). Fast gapped-read alignment with Bowtie 2. Nat Methods.

[34] Tarazona S (2015). Data quality aware analysis of differential expression in RNA-seq with NOISeq R/Bioc package. Nucleic Acids Res..

[35] Kanehisa M, Goto S, Sato Y, Furumichi M, Tanabe M (2012). KEGG for integration and interpretation of large-scale molecular data sets. Nucleic Acids Res..

[36] Dysvik B, Jonassen I (2001). J-Express: exploring gene expression data using Java. Bioinformatics.

[37] Weltzien FA (2003). Identification and localization of eight distinct hormone-producing cell types in the pituitary of male Atlantic halibut (Hippoglossus hippoglossus L.). Comp Biochem Physiol A Mol Integr Physiol.

[38] Wargelius A (2016). Dnd knockout ablates germ cells and demonstrates germ cell independent sex differentiation in Atlantic salmon. Sci Rep.

[39] Lien S (2016). The Atlantic salmon genome provides insights into rediploidization. Nature.

[40] Edgar RC (2004). MUSCLE: multiple sequence alignment with high accuracy and high throughput. Nucleic Acids Res..

[41] Li H (2009). The Sequence Alignment/Map format and SAMtools. Bioinformatics.

[42] Altschul SF, Gish W, Miller W, Myers EW, Lipman DJ (1990). Basic local alignment search tool. J Mol Biol.

[43] Miura C, Miura T, Kudo N, Yamashita M, Yamauchi K (1999). cDNA cloning of a stage-specific gene expressed during HCG-induced spermatogenesis in the Japanese eel. Dev Growth Differ.

[44] Pfennig F (2012). The social status of the male Nile tilapia (Oreochromis niloticus) influences testis structure and gene expression. Reproduction.

[45] Melo MC (2015). Androgens directly stimulate spermatogonial differentiation in juvenile Atlantic salmon (Salmo salar). Gen Comp Endocrinol.

[46] Zhou Q, Li L, Zhao B, Guan KL (2015). The hippo pathway in heart development, regeneration, and diseases. Circ Res.

[47] Christensen, K. A., Gutierrez, A. P., Lubieniecki, K. P. & Davidson, W. S. TEAD3, implicated by association to grilsing in Atlantic salmon. *Aquaculture*, 10.1016/j.aquaculture.2017.06.026 (2017).

[48] Zhao B (2007). Inactivation of YAP oncoprotein by the Hippo pathway is involved in cell contact inhibition and tissue growth control. Genes Dev.

[49] Kleppe L, Wargelius A, Johnsen H, Andersson E, Edvardsen RB (2015). Gonad specific genes in Atlantic salmon (Salmon salar L.): characterization of tdrd7-2, dazl-2, piwil1 and tdrd1 genes. Gene.

[50] Schulz RW (2010). Spermatogenesis in fish. Gen Comp Endocrinol.

[51] França, L. R., Nóbrega, R. H., Morais, R. D. V. S., De Castro Assis, L. H. & Schulz, R. W. In *Sertoli Cell Biology* (*Second Edition*) 385–407 (Academic Press, 2015).

[52] Leal MC (2009). Histological and stereological evaluation of zebrafish (Danio rerio) spermatogenesis with an emphasis on spermatogonial generations. Biol Reprod.

[53] van den Hurk R, Peute J, Vermeij JA (1978). Morphological and enzyme cytochemical aspects of the testis and vas deferens of the rainbow trout, Salmo gairdneri. Cell Tissue Res.

[54] Lubzens E, Young G, Bobe J, Cerda J (2010). Oogenesis in teleosts: how eggs are formed. Gen Comp Endocrinol.

[55] Trombley S, Mustafa A, Schmitz M (2014). Regulation of the seasonal leptin and leptin receptor expression profile during early sexual maturation and feed restriction in male Atlantic salmon, Salmo salar L., parr. Gen Comp Endocrinol.

[56] Larsen DA (2006). Growth modulation alters the incidence of early male maturation and physiological development of hatchery-reared spring Chinook salmon: A comparison with wild fish. Trans Am Fish Soc.

[57] Silverstein JT, Shearer KD, Dickhoff WW, Plisetskaya EM (1999). Regulation of nutrient intake and energy balance in salmon. Aquaculture.

[58] Silverstein JT, Shearer KD, Dickhoff WW, Plisetskaya EM (1998). Effects of growth and fatness on sexual development of chinook salmon (Oncorhynchus tshawytscha) parr. Can J Fish Aquat Sci.

