# Supplementary material for: Vgll3 and the Hippo pathway are regulated in Sertoli cells upon entry and during puberty in Atlantic salmon testis
Source: Sci Rep. 2018 Jan 30;8:1912. doi: 10.1038/s41598-018-20308-1 (PMC5789820; doi:10.1038/s41598-018-20308-1)
Supplement: Supplementary file 1 — Supplementary information [file 41598_2018_20308_MOESM1_ESM.pdf]

Supplementary Information for

Vgll3 and the Hippo pathway are regulated in Sertoli cells upon entry and during puberty in Atlantic salmon testis

Erik Kjærner-Semb (erikkj@imr.no)[1,2,#], Fernando Ayllon (fernando.ayllon@imr.no)[1], Lene Kleppe (lene.kleppe@imr.no)[1], Elin Sørhus (elin.sorhus@imr.no)[1], Kai Skaftnesmo (kai.ove.skaftnesmo@imr.no)[1], Tomasz Furmanek (tomasz.furmanek@imr.no)[1], Frida T. Segafredo (frida_19_92@hotmail.com)[1], Anders Thorsen (anders.thorsen@imr.no)[1], Per Gunnar Fjelldal (pergf@imr.no)[3], Tom Hansen (tomh@imr.no)[3], Geir Lasse Taranger (geirt@imr.no)[1], Eva Andersson (eva.andersson@imr.no)[1], Rüdiger W. Schulz (R.W.Schulz@uu.nl)[1,4,] , Anna Wargelius (anna.wargelius@imr.no)[1]*, Rolf B. Edvardsen (rolf.brudvik.edvardsen@imr.no)[1]*

*shared senior authorship

[1] Institute of Marine Research, P.O. Box 1870, Nordnes, NO-5817 Bergen, Norway

[2] Department of Biology, University of Bergen, Bergen, Norway

[3]Institute of Marine research, Matre Aquaculture Research Station, 5984 Matredal, Norway

[4] Utrecht University, Science Faculty, Department Biology, Padualaan 8, NL-3584 CH Utrecht, The Netherlands.

Contact

[#]Corresponding author, erikkj@imr.no

This file includes

Supplementary Figures S1 to S6

Supplementary Tables S1 and S2

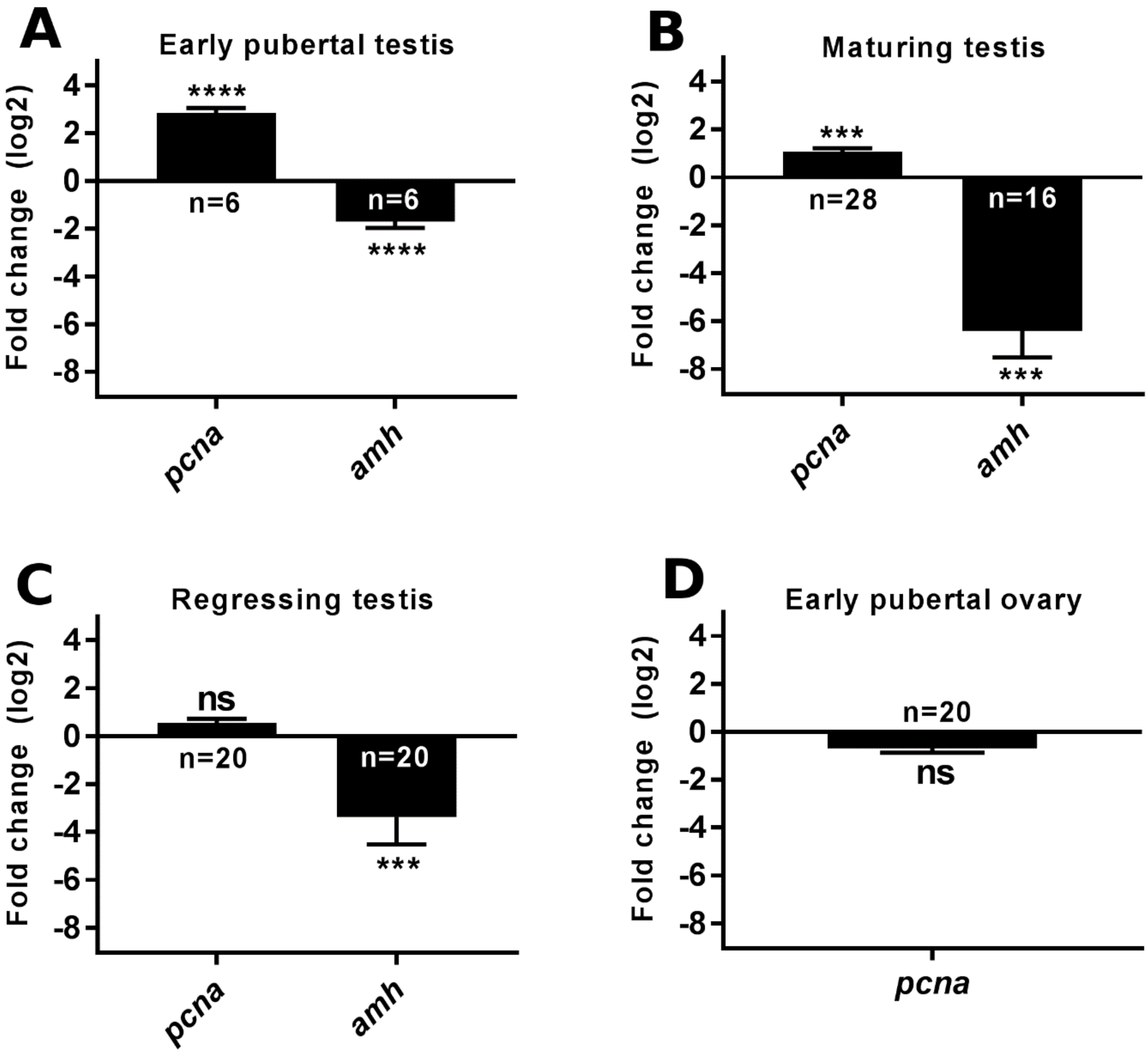

Supplementary Fig S1 – Expression of *pcna* and *amh*. A) Relative expression (fold change) in early pubertal testes relative to prepubertal testes, based on RNA-Seq data. **B**) Relative expression in maturing testis compared to prepubertal testis based on qPCR. **C**) Relative expression in testis regressing from maturity relative to prepubertal testis measured with qPCR. **D**) Relative expression in early pubertal relative to prepubertal ovary, measured with qPCR. Error bars show SEM. Total number of individuals assayed is shown in each box, of which half are prepubertal control group. ns = not significant, *** = P < 0.001, **** = P < 0.0001.

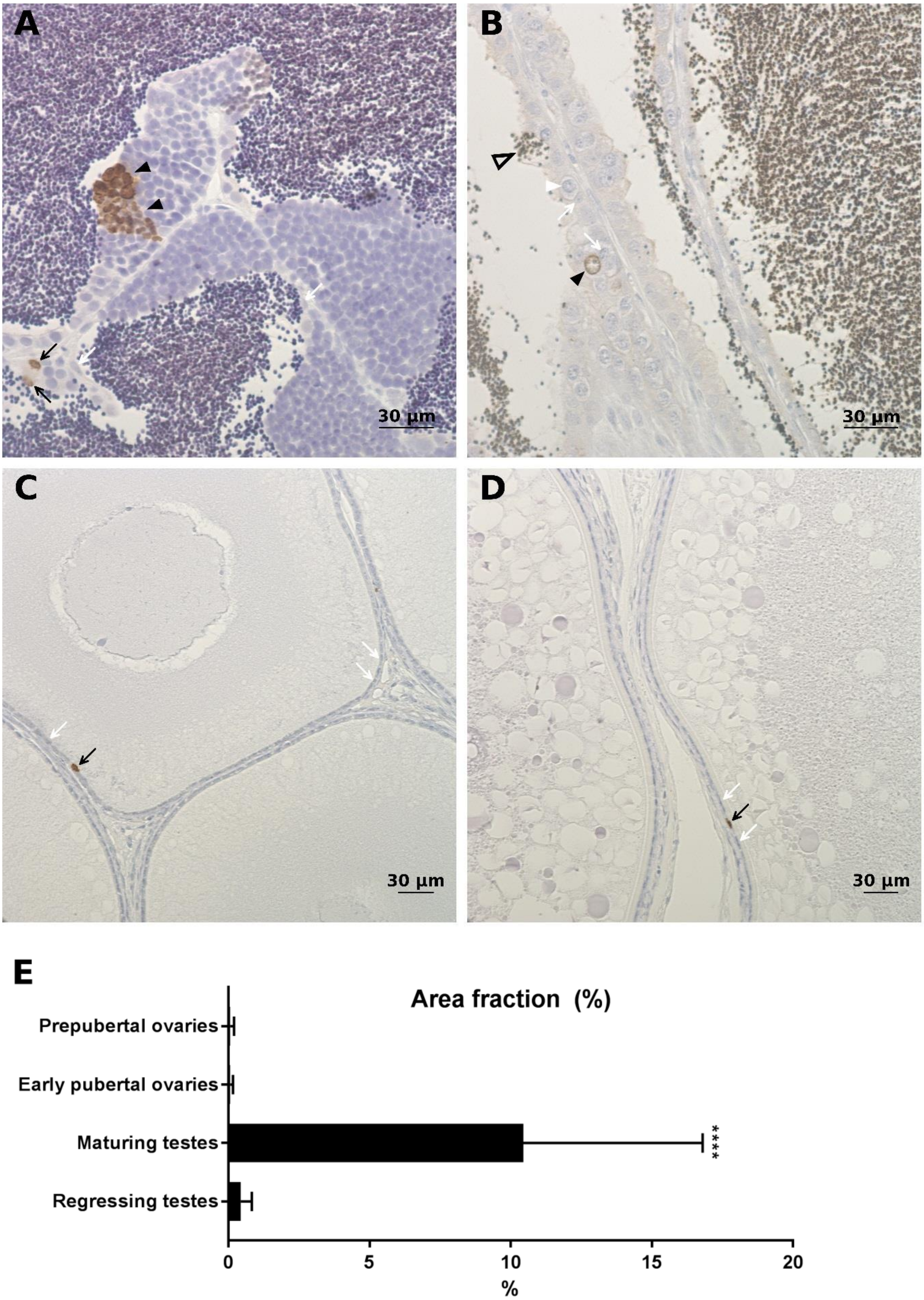

Supplementary Fig S2 – Proliferation activity in gonads. Immunocytochemical localization of the proliferation marker phosphorylated histone H3 (pH3). **A)** Maturing testis from Male experiment 2, **B)** testis regressing from maturity from Male experiment 3, **C)**

prepubertal ovary and **D)** early pubertal ovary from the Female experiment. The scale bars indicate 30 µm. Black arrows indicate dividing Sertoli cells (**A**) and dividing granulosa cells (**C** and **D**). White arrows indicate non-dividing Sertoli cells (**A** and **B**) and non-dividing granulosa cells (**C** and **D**). Black arrowheads indicate proliferating germ cell(s). Open black arrowhead indicates removal of sperm by Sertoli cells. **E**) Area fractions of pH3-positive cells in different pubertal stages of male and female gonads (n=3 individuals per stage). Error bars show SEM. **** = $P < 0.0001$.

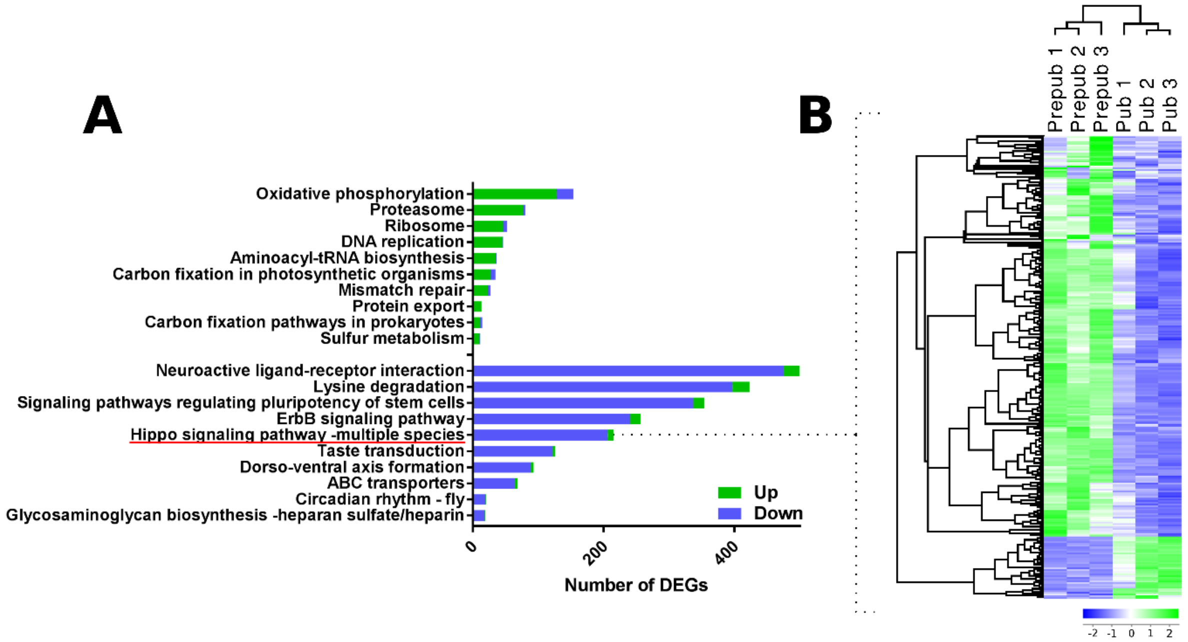

Supplementary Fig S3 - Regulation of pathways. A) The ten most up and down-regulated KEGG pathways in the transition from the prepubertal to the pubertal stage. Bars show number of significant ($P < 0.05$) DEGs (FC >1.5) that were up-regulated (green) and down-regulated (blue) in pathways with ≥ 10 DEGs. **B**) Expression of all KEGG Hippo pathway genes in prepubertal and pubertal testis from Male experiment 1, showing genes significantly different ($P < 0.05$, FC > 1.5) between the two stages. Of the genes that were up-regulated in pubertal stage, 37% (n=14) were *ywha* genes. For gene names and normalized read counts see Supplementary File S1.

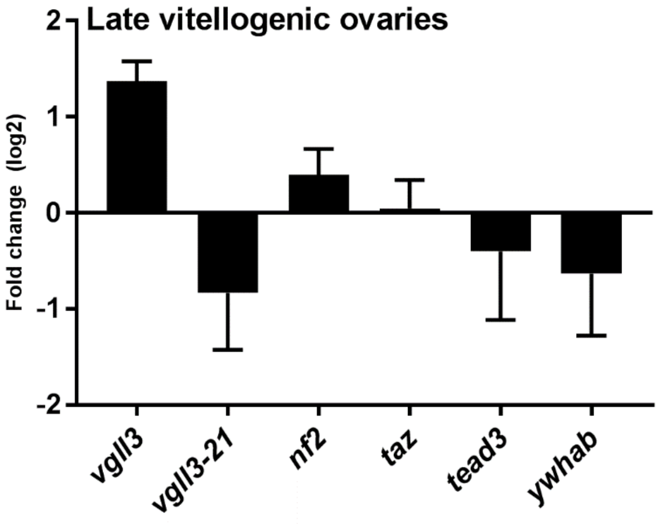

Supplementary Fig S4 - Expression of Hippo genes in late vitellogenic ovaries. Expression of selected Hippo pathway genes were analyzed in ovaries from oil drop (Od) and late vitellogenic (Ty) stages. The fish had been reared under natural light conditions (NL), with stages further described in Andersson et al., 2013[1]. The analysis included n=5-6 fish from each stage.

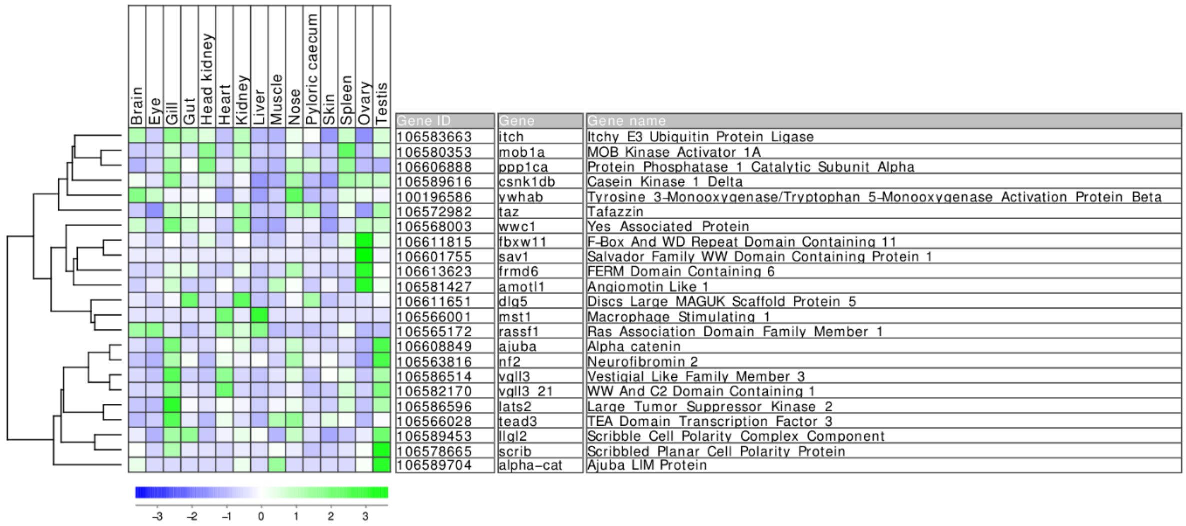

Gene ID	Gene	Gene name
106583663	itch	Itchy E3 Ubiquitin Protein Ligase
106580353	mob1a	MOB Kinase Activator 1A
106606888	ppp1ca	Protein Phosphatase 1 Catalytic Subunit Alpha
106589616	csnk1db	Casein Kinase 1 Delta
100196586	ywhab	Tyrosine 3-Monooxygenase/Tryptophan 5-Monooxygenase Activation Protein Beta
106572982	taz	Tafazzin
106568003	wwc1	Yes Associated Protein
106611815	fbxw11	F-Box And WD Repeat Domain Containing 11
106601755	sav1	Salvador Family WW Domain Containing Protein 1
106613623	frmd6	FERM Domain Containing 6
106581427	amotl1	Angiomotin Like 1
106611651	dlg5	Discs Large MAGUK Scaffold Protein 5
106566001	mst1	Macrophage Stimulating 1
106565172	rassf1	Ras Association Domain Family Member 1
106608849	ajuba	Alpha catenin
106563816	nf2	Neurofibromin 2
106586514	vgll3	Vestigial Like Family Member 3
106582170	vgll3 21	WW And C2 Domain Containing 1
106586596	lats2	Large Tumor Suppressor Kinase 2
106566028	tead3	TEA Domain Transcription Factor 3
106589453	llgl2	Scribble Cell Polarity Complex Component
106578665	scrib	Scribbled Planar Cell Polarity Protein
106589704	alpha-cat	Ajuba LIM Protein

Supplementary Fig S5 - Expression of Hippo genes in different tissues. Expression of selected Hippo pathway genes were analyzed in RNA-seq data available on SRA (BioProject ID PRJNA72713). Regulation of these in testis maturation is shown in Fig 2.

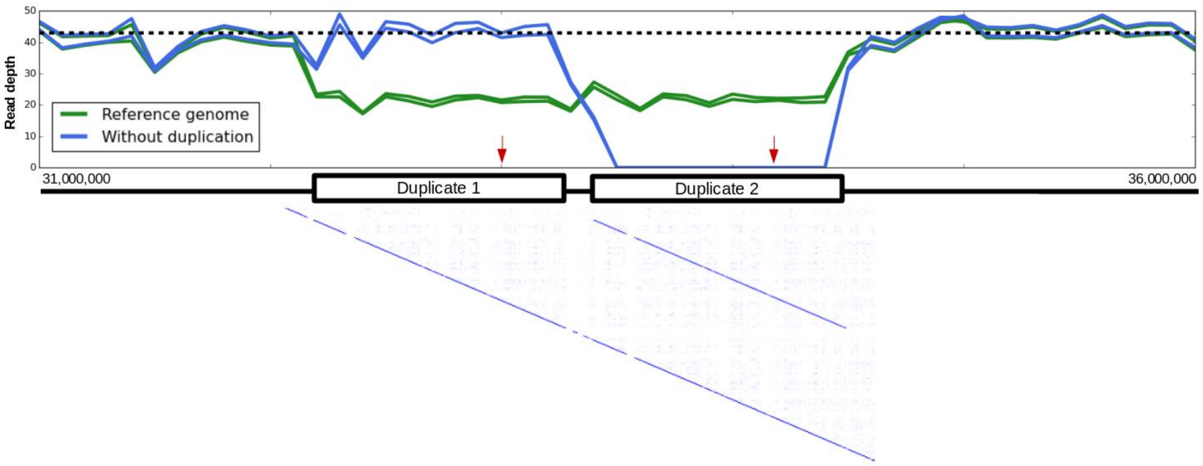

Supplementary Fig S6 - Correction of *vgll3* duplication artifacts. In the Atlantic salmon reference genome, a 1 MB region on Chr 21 containing *vgll3*$_{21}$ has been duplicated (pos 32,160,000-33,230,000 and 33,350,000-34,420,000), most likely as a result of an error in the genome assembly process. In the top plot, genomic sequence data from two different Atlantic salmon populations (BioProject ID PRJNA293012, Suldalslågen and Eidselva) was mapped to the reference genome (green line). Sequences mapping to Chr 21 were remapped to a version of the chromosome where the second copy of the duplicated region was removed (blue lines). Depth of coverage is shown in 100 kb windows, using only properly paired reads. Average depth of coverage in Chr 21 is shown as a black dashed line. Location of *vgll3*$_{21}$ is indicated with red arrows. The X-axis shows genomic positions. Removing the second duplicate and remapping the sequence data results in doubled depth of coverage in the first duplicate, at a level similar to the chromosome average. The bottom plot shows BLAST scores where 10 kb windows were aligned against each other, and where the second duplicate is observed as an additional blue diagonal line. Further, three copies of *vgll3*$_{21}$ were found in short unplaced contigs (not assigned to chromosomes) in the reference genome. When aligned to *vgll3*$_{21}$, these showed 100% sequence similarity, and are also believed to be a result of errors in the genome assembly process.

Supplementary Table S1 - Primers used for qPCR.

Gene	Gene ID	Chr	Slope	R^2	Forward primer	Reverse primer
elf1a[#]	100196887	14	-3.37	0.997	GCCCCTGCAGGACGTCTA	CGGCCCACGGGTACTGT
scamp1[#]	106563043	11	-3.50	0.983	CGGACCCTGAATTCAATAACCC	GTGGGGGCCGGTACTTTAG
nf2	106563816	11	-3.44	0.988	ACCACCCTACATGCCCCATA	CCCCTGTTTTCTCCAATGGC
tead3	106566028	12	-3.25	0.976	CCCACAGAACGCCTTTTTCC	GTACTGGCTGCTGACTCCAT
taz	106572982	15	-3.22	0.982	AGACCTTCTGGAGTCACTACG	GGAATCAGTCAGGGCCTTCC
vgll3	106586514	25	-3.38	0.995	ATGTGGTGGATGAGCATTTCTC	TTGGTCTCTCCGGTGAAGGT
vgll3$_{21}$	106582170	21	-3.13	0.990	GCCTGGCCCGCAACTCT	CCTCTCTCAGAACCAAACAGTATTGA
ywhab	100196586	1	-3.40	0.993	ATACCGGGAGAAGGTGGAGG	GCTTTCAGCATTTGTGGAGTT
pcna	106585071	24	-3.30	0.997	CAAGGAGGAGGAAGCTGTGAC	GAAGTTAAGGTAGTTCAGTGCG
amh	100136452	10	-3.55	0.997	CAGTCACTCTCTGCAGCCTTACAA	CAACATTGAATCTCCATTTCAGTTTAC

[#] Used as endogenous control

Supplementary Table S2 – Primers used for generating probe for *in situ* Hybridization with *vgll3*. Sp6 and T7 sequences are marked with gray background. The probe is 542 bp.

Name	Primer sequence
Sp6 Fwd	ATTTAGGTGACACTATAGGGAAGGCCAGTGCAGTTCTC
Sp6 Rev	TCCATGGGTGTGTGTAGAGC
T7 Fwd	GGAAGGCCAGTGCAGTTCTC
T7 Rev	TAATACGACTCACTATAGGGTCCATGGGTGTGTGTAGAGC

SUPPLEMENTARY REFERENCES

1 Andersson, E. *et al.* Pituitary gonadotropin and ovarian gonadotropin receptor transcript levels: seasonal and photoperiod-induced changes in the reproductive physiology of female Atlantic salmon (Salmo salar). *Gen Comp Endocrinol* **191**, 247-258, doi:10.1016/j.ygcen.2013.07.001 (2013).
